# The Primacy of Public Health Considerations in Defining Poor Quality Medicines

**DOI:** 10.1371/journal.pmed.1001139

**Published:** 2011-12-06

**Authors:** Paul N. Newton, Abdinasir A. Amin, Chris Bird, Phillip Passmore, Graham Dukes, Göran Tomson, Bright Simons, Roger Bate, Philippe J. Guerin, Nicholas J. White

**Affiliations:** 1 Wellcome Trust-Mahosot Hospital-Oxford Tropical Medicine Research Collaboration, Microbiology Laboratory, Mahosot Hospital, Vientiane, Lao PDR; 2 Centre for Tropical Medicine, Nuffield Department of Medicine, University of Oxford, Churchill Hospital, Oxford, United Kingdom; 3 WorldWide Antimalarial Resistance Network (WWARN), University of Oxford, Oxford, United Kingdom; 4 Malaria & Child Survival Department, Population Services International, Nairobi, Kenya; 5 Wellcome Trust, London, United Kingdom; 6 School of Pharmacy, Curtin University of Technology, Bentley, Western Australia, Australia; 7 University of Oslo, Oslo, Norway; 8 IHCAR Div Global Health, Karolinska Institute, Stockholm, Sweden; 9 IMANI Center for Policy & Education, Accra, Ghana; 10 American Enterprise Institute, Washington, D.C., United States of America; 11 Mahidol Oxford Research Unit, Faculty of Tropical Medicine, Mahidol University, Bangkok, Thailand

## Abstract

Paul Newton and colleagues argue that public health, and not intellectual property or trade issues, should be the prime consideration in defining and combating counterfeit medicines, and that the World Health Organization (WHO) should take a more prominent role.

**Summary Points**

- Poor quality essential medicines, both substandard and counterfeit, are serious but neglected public health problems. Anti-infective medicines are particularly afflicted.
- Unfortunately, attempts to improve medicine quality have been hampered by confusion and controversy over definitions. For counterfeit (or falsified) medicines, this has arisen from perceived differences between public health and intellectual property approaches to the problem.
- We argue that public health, and not intellectual property or trade issues, should be the prime consideration in defining and combating counterfeit medicines, and that the World Health Organization (WHO) should be encouraged and supported to take a more prominent role in improving the world's medicine quality and supply.
- An international treaty on medicine quality, under WHO auspices, could be an important step forward in the struggle against both substandard and counterfeit (or falsified) medicines.

## Poor Quality Medicines—A Major Public Health Problem

There is growing, but belated, concern that much of the developing world's supply of medicines—in particular, its supply of anti-infective drugs—is of poor quality. This constitutes a major public health problem because the high prevalence of poor quality drugs in developing countries results in avoidable morbidity, mortality, and drug resistance [1]–[7]. Moreover, any efforts to improve public health by developing new medicines or by changing treatment policies will ultimately be pointless if the drugs patients actually take contain insufficient or incorrect ingredients.

Unfortunately, efforts to improve the quality of medicines in developing countries are being hampered by confusion over the terms used to describe different types of poor quality medicines. This confusion has arisen because of poor science and because of tension between the defence of commercial interests and the public health importance of enhanced access to good quality medicines in developing countries. Specifically, some commentators have argued that counterfeit medicines are being viewed primarily as intellectual property (IP) rather than public health concerns and that the innovative pharmaceutical industry is using action against counterfeit medicines to impede the trade in competing generics [8]–[20]. In this essay, we call for public health concerns to be made the prime consideration in defining and combating counterfeit medicines and argue that recent World Health Organization (WHO) initiatives eschew IP concerns. We also discuss some related but neglected interventions that might help to improve drug quality in developing countries.

## Current Definitions of Poor Quality Medicines

Since 1992, the WHO has used a definition of counterfeit medicines that regards them as products produced fraudulently without any regard to regulatory and public health concerns (Box 1) and usually, but not always, lacking in any active pharmaceutical ingredients (API) [3]–[5],[9]. By contrast, the definition used by WHO for “substandard” medicines describes them as medicines produced by legitimate manufacturers that do not meet pharmacopoeial standards because of errors in the quality or quantity of raw materials or in manufacturing (see Box 1; [7]).

**Box 1. Current Definitions as used by WHO**

**Substandard medicines**

*“Substandard medicines (also called out of specification (OOS) products) are genuine medicines produced by manufacturers authorized by the NMRA which do not meet quality specifications set for them by national standards.*

“*Normally, each medicine that a manufacturer produces has to comply with quality standards and specifications. These are reviewed and assessed by the national medicines regulatory authority before the product is authorized for marketing.*” [34]

**Counterfeit medicines**

“*A counterfeit medicine is one which is deliberately and fraudulently mislabelled with respect to identity and/or source. Counterfeiting can apply to both branded and generic products and counterfeit products may include products with the correct ingredients, wrong ingredients, without active ingredients, with insufficient quantity of active ingredient or with fake packaging*.” [9]

Surprisingly, some commentators argue that it is “not immediately obvious that a specific definition of ‘counterfeit medicine’ is a necessary tool to effectively combat the public health problem of unsafe medicines” [8]. We strongly disagree with this viewpoint. It is clearly crucial to distinguish counterfeits from other types of poor quality medicines, in particular, substandard medicines. Counterfeit and substandard medicines are fundamentally different problems and without common understanding, through consensus definitions, the public health problems associated with poor quality medicines cannot be measured and interventions planned and evaluated (see Text S1).

**Figure 1:**
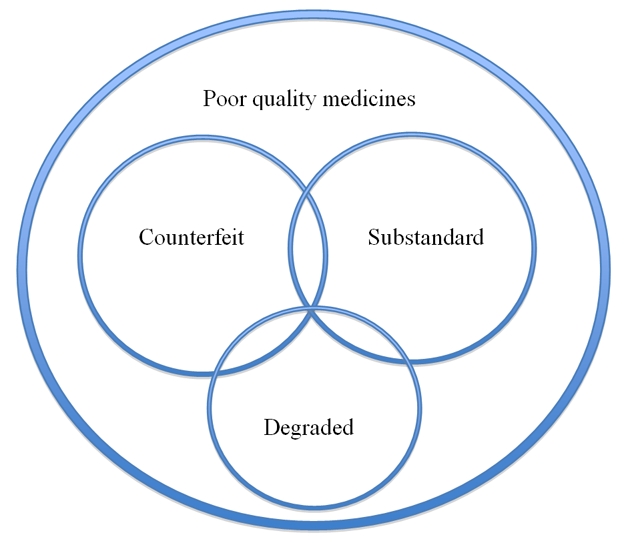
A Venn diagram illustrating public health–oriented definitions of poor quality medicines. “Poor quality medicines” is a term inclusive of counterfeit, substandard, and degraded medicines and also for medicines that fail chemistry analysis but with insufficient information to determine whether they are counterfeit, substandard, or degraded. The available data do not allow relative sizing of the area of each circle in proportion to the frequency of type of poor quality medicine. There could be grey areas between all three main types (see Text S1). For example, both substandard medicines and counterfeits could become degraded after manufacture.

Unfortunately, although it is essential to distinguish substandard from counterfeit medicines because their origins and solutions differ, these terms are often used interchangeably, which is both confusing and incorrect [3],[5],[8] (Box 1 and Text S1). For example, “poor quality medicines”—drugs that have failed physical/chemical tests—are often classified as “counterfeit” even when it is unclear whether they have been produced fraudulently (i.e., they are counterfeit) or whether they are the result of poor quality control (i.e., they are substandard) (Figure 1). The problem of substandard medicines can potentially be remedied through action by medicine regulatory authorities (MRAs), through initiatives such as WHO prequalification, and through support for improvement of factory processes. By contrast, the remedies for counterfeits include covert MRA, police, and customs investigations, “factory” closures, prosecutions, and the reduction of the profit incentive of counterfeiting by ensuring that genuine medicines are affordable and accessible [21].

## A New Definition for Counterfeit Medicines

An important unresolved issue with existing definitions of counterfeit medicines is the tension between the implicit or explicit understanding that counterfeit, unlike substandard, medicines are produced intentionally and the difficulty, in law, of proving that a counterfeit medicine has been manufactured “deliberately” with an intention to mislead. With the current definition, prosecutors have to show both that the manufacture of a “counterfeit” medicine actually took place and that the manufacturer had the intention to mislead. To help MRAs combat the problem of counterfeit drugs, WHO [9] and its partners in the International Medicinal Products Anti-Counterfeiting Taskforce (IMPACT) recently proposed a refinement to the 1992 definition of counterfeit medicines to provide a model text for national legislation (Box 2; [8],[9]). Some agree with this refinement, but others have argued that it defines counterfeit medicine from an IP perspective and could therefore damage access to generic medicines. While we agree that any public health–oriented definition of counterfeit medicines should not invoke IP issues, we argue that the proposed WHO definition does **not** invoke such issues [10]–[20]. Importantly, there has been little discussion of alternative non-IP legislation that could be used to prevent the circulation of counterfeit medicines [1]–[6],[10],[20].

**Box 2. Proposed New Definitions**

**Substandard medicines**

“*Each pharmaceutical product that a manufacturer produces has to comply with quality standards and specifications at release and throughout the product shelf-life required by the territory of use. Normally, these standards and specifications are reviewed, assessed and approved by the applicable National Medicines Regulatory Authority before the product is authorized for marketing.*

*“Substandard medicines are pharmaceutical products that do not meet their quality standards and specifications.*” [35]

**Counterfeit medical products**

“*A medical product is counterfeit when there is a false representation in relation to its identity and/or source. This applies to the product, its container or other packaging or labeling information. Counterfeiting can apply to both branded and generic products. Counterfeits may include products with correct ingredients/components, with wrong ingredients/components, without active ingredients, with incorrect amounts of active ingredients, or with fake packaging.*

“*Violations or disputes concerning patents must not be confused with counterfeiting of medical products. Medical products (whether generic or branded) that are not authorized for marketing in a given country but authorized elsewhere are not considered counterfeit. Substandard batches or quality defects or non-compliance with Good Manufacturing Practices/Good Distribution Practices in legitimate and medical products should not be confused with counterfeiting.*” [9] (Footnotes omitted.)

And gave the following explanation:

“*Many Member States do not have specific or effective legal instruments for combating counterfeit medical products, and may for that reason resort to non-specific legislation related to trademark protection. However, for several reasons such an approach is not satisfactory, as follows. Legal instruments related to intellectual property rights have a broad scope and are not focused on the protection of public health. Counterfeiting of medical products does not always entail the violation of intellectual property rights. The intellectual property rights approach identifies the rights holder as the main victim of counterfeiters and as the main trigger of enforcement and prosecution while, in the case of medical products, the real victim of counterfeiting is the patient; legislation should therefore enable patients and health authorities to undertake appropriate procedures regardless of the action of the holders of intellectual property rights. The technical complexity of the regulation of manufacture, trade, distribution and dispensing of medical products warrants an approach much wider than one based on intellectual property rights. The new text therefore states clearly that the violations or disputes about patents must not be confused with counterfeiting of medical products*.” [9]

## A Public Health Perspective Definition of Counterfeit Medicines

Although some commentators have expressed the view that counterfeit medicines are not a public health issue, despite the evidence to the contrary [1]–[7],[22], we believe that a logical response to the above concerns about definitions would be a robust public health–oriented definition of counterfeit medicines designed to protect access to good quality medicines.

Importantly, it is not only innovator medicines that are counterfeited (we prefer the term “innovator medicines” to “branded medicines” in this context because generic medicines can also be branded). Generic medicines, which are of enormous benefit to global public health because they make essential medicines less expensive and more accessible, are also counterfeited; and so any definition of counterfeited medicines must be appropriate for both innovative and generic drugs. In fact, contrary to the opinion of some [6],[10],[12]–[20], the new WHO definition of counterfeit medicines (Box 2) is less likely to damage access to generic drugs than the 1992 version (see Text S1), and it distances itself from patent issues, as WHO states explicitly (Box 3; [9]).

**Box 3. Separating the Issue of Counterfeit Medicines from Patent Issues**

A report by the WHO Secretariat on counterfeit medical products includes the following draft resolution:

“***14.*** *The Executive Board is invited to consider the following draft resolution:*

*The Executive Board,*

*Having considered the report on counterfeit medical products,*

*RECOMMENDS to the Sixty-second World Health Assembly the adoption of the following resolution:…….*

*Recognizing that the primary focus of combating counterfeit medical products is the protection of public health and that the main victims of counterfeiters are patients;*

*Recognizing the importance of ensuring that combating counterfeit medical products does not result in hindering the availability of legitimate generic medicines;*

*Recognizing that disputes about, or violations of, patents are not to be confused with counterfeiting;*

*Recognizing that medical products (whether generic or branded) that are not authorized for marketing in a given country but authorized elsewhere are not considered counterfeit;*

*Recognizing that quality defects or non-compliance with Good Manufacturing Practices/Good Distribution Practices in legitimate medical products must not be confused with counterfeiting;*”

([9], with corrigendum.)

However, in addition to the potential that IP-based definitions of counterfeit medicines have for hampering the trade in generics, such a perspective is inappropriate for a public health problem for three more reasons. First, in stark contrast to the counterfeiting of consumer goods, such as DVDs, the primary victims of medical counterfeiting are not the legitimate manufacturers but patients, and often the poorest and most vulnerable of patients. We believe that patients deserve to be protected under unequivocal public health–oriented law rather than being subject to the uncertainties of contentious IP law. Second, crucially, allegations of trademark or patent violations will not protect the public adequately against counterfeit medicines that are labeled as manufactured by non-existent companies because a non-entity cannot be sued. For example, counterfeit tetracycline tablets that contained no API were recently found in Cambodia. These tablets were labeled as made by a non-existent company with no patents or trademark rights and therefore no viable claims under IP law could be made against this medication [22]. Third, IP law cannot necessarily be used to protect unbranded generic medicines against the production of counterfeit medicines.

A further controversial problem is the issue of whether “counterfeit” is the best term for this particular class of poor quality medicines. There has been much discussion as to whether the correct term for counterfeit medicine should be “counterfeit”, “fake”, “falsified”, or “spurious” medicine, as it is claimed that using “‘counterfeit’ (a term defined in the TRIPS Agreement as trademark violation) to also refer to spurious pharmaceuticals is a disservice to public health as it conflates issues of health and IPRs” [20],[23],[24]. Which of the above terms is used for what we refer to as “counterfeit medicine” throughout this essay is of relatively little importance in comparison to consensus on the accompanying definition, which is vital. If the use of an alternative term, such as “falsified” or “spurious”, creates the conditions for constructive vital dialogue, such a change in terminology should be enthusiastically embraced. Further neglected issues concerning the definitions of different types of poor quality medicines are discussed in Text S1.

## The World Health Assembly's Failure to Deal with Counterfeit Medicines

The World Health Assembly (WHA) should be an ideal setting in which to thrash out solutions to the problem of poor quality medicines in the developing world. However, two days of debate on counterfeit medicines at the 63rd WHA (May 2010) failed to reach any agreement about the way forward apart from establishment of a working group to “examine, from a public health perspective, excluding trade and intellectual property considerations…WHO's role in measures to ensure availability of quality, safe, efficacious and affordable medical products…WHO's relationship with the International Medicinal Products Anti-Counterfeiting Taskforce…WHO's role in the prevention and control of medical products of compromised quality, safety and efficacy such as substandard/spurious/falsely-labelled/falsified/counterfeit medical products from a public health perspective….” [23]–[28]. The Working Group met at the 64th WHA in May 2011 and requested more time for its deliberations [29]. This delay is not in the interest of global public health, and we hope that a consensus on counterfeit medicine definitions, terminology, and interventions will soon be expedited by MRAs and public health officials working together, as urged by 40 African countries [26] whose voices reflect public health concerns and whose people are most likely to suffer from this stagnation. The Working Group met, for the second time, on October 25–28, 2011. The report is available at http://www.ip-watch.org/weblog/wp-content/uploads/2011/11/SSFFCReport_28OCT.pdf.

## The Way Forward

The problems of poor quality medicines cannot be viewed in isolation, as they are enmeshed with many other complex health system problems, especially the affordability and accessibility of medicines and the (often limited) capacity of MRAs. Here, we highlight three potential interventions that might help to deal with poor quality medicines; many other parallel interventions will be needed to solve this problem, few of which have been, as yet, planned or enacted [1]–[5],[7].

First, it is very important for public health that the generics industry is protected from the inappropriate use of IP law to stifle competition, but at the same time it is vital that more is done to combat the disastrous public health consequences of poor quality medicines. Access to medicines and medicine quality are two sides of the same coin. Importantly, both innovative and generic drug industries can experience conflicts of interest in relation to public health, as they are both for-profit enterprises [30]. We believe, therefore, that public health–oriented definitions should help improve the overall quality of the world's medicine supply by both creating common understanding on poor quality medicines and facilitating the protection of trade in generics.

Second, we suggest that the current situation could potentially be helped and trust increased if organizations with potential conflicts of interests and IP perspectives issued unambiguous statements eschewing the use of IP law to counter generic medicines. For example, the director general of the International Federation of Pharmaceutical Manufacturers & Associations (IFPMA) has done this by recently stating that: “We have no agenda to interfere with the legitimate trade in generics. Heightened efforts by high income country governments to fight fake products of all kinds are not a pretext for imposing high IP standards on others that may not be ready for them” [31] and “A medicine that is authorized for marketing by one regulatory authority but not by another should not be regarded as counterfeit on these grounds alone in the latter's territory” [32] and called for WHO leadership in this matter [31],[32].

Third, a treaty on medicine quality, drafted under the auspices of the WHO, has recently been suggested as a way forward [33]. This could be negotiated incrementally, starting from the agreement by all parties that poor quality medicines are unacceptable, with emphasis on reaching a consensus on the contentious points, such as definitions and terms. Such a treaty could facilitate transnational jurisdiction over widespread, systematic counterfeiting as a crime against humanity. It could also include positive legal powers and a financial mechanism to facilitate good quality manufacturing and access to affordable high quality medicines to reduce the frequency of substandard medicines (A. Attaran, personal communication).

## Conclusions

Counterfeit medicines should be defined in terms of harm to health, with punishments appropriate for the injury or killing of patients. Moreover, it is imperative that public health institutions, ministries, and lawyers, and not primarily IP specialists or industrial and trade bodies, take the strategic lead in countering poor quality medicines. We strongly suggest that those concerned with medicine quality and access put the recent controversies behind them and work positively towards agreement on definitions and a treaty to facilitate access to good quality essential medicines and medical products.

## Supporting Information

Text S1 **Some further problematic issues relating to medicine quality.**
(DOC)

## Abbreviations

IP: intellectual property
MRA: medicine regulatory authority
WHO: World Health Organization
