# Supplementary material for: The Primacy of Public Health Considerations in Defining Poor Quality Medicines
Source: PLoS Med. 2011 Dec 6;8(12):e1001139. doi: 10.1371/journal.pmed.1001139 (PMC3232210; doi:10.1371/journal.pmed.1001139)
Supplement: Text S1 — Some further problematic issues relating to medicine quality. (DOC) [file pmed.1001139.s001.doc]

**Supporting Information S1**

**The scale of the problem: how common are poor quality medicines?**

The term ‘counterfeit’ is frequently used as a general term for poor quality medicines in much of the lay and scientific press, which has led to a great deal of confusion. In 2010, a group of NGOs submitted an Open Letter to the Director-General of WHO [25], stating that ‘*confusion over the use of the term “Counterfeit” makes it impossible to obtain data on the true extent of the proliferation of medicines which do not meet quality….*’ and ‘*… empirical, reliable and transparent statistics about “counterfeit drugs” is non-existent…’*. We disagree with this statement. Although data on the frequency and consequences of poor quality medicines are relatively sparse, they are consistent, and they strongly support the contention that both counterfeit and substandard medicines are major public health problems [3-5,7,22,36-41]. However, it is clear that more data will be required to provide accurate estimates of the prevalence of poor quality drugs and to understand the relationship between poor quality drugs and public health. Thus, initiatives to provide the public health community with reliable contemporaneous data on the global epidemiology of poor quality medicines are needed. We believe that a revised definition of counterfeit medicines would facilitate collection of such objective data and allow better understanding of the comparative epidemiology of counterfeit and substandard medicines. By analogy, until recently, estimates of malaria mortality were unreliable. Fortunately, this situation did not prevent long term efforts to reduce malaria incidence through global initiatives whilst more reliable statistics were being collected.

**Problems with IP-based definitions for counterfeit medicines and access to generic drugs**

Two recent examples illustrate clearly the problem of misusing IP laws when applied to medicines.

First, proposed changes to laws in some East African countries include definitions of counterfeit medicines that could lead to good quality genuine generic medicines being classed as counterfeit (because of a patent or trademark dispute). This is likely to harm public health by reducing patients’ access to affordable medicines [42-43]. Although the use by WHO of the term ‘counterfeit medicine’ has been linked to these legislative changes [25], as far as we are aware there is no published objective evidence of such linkage. However, a robust, internationally agreed public health-orientated definition of counterfeit medical products would reduce the risk of countries making diverse and dangerous definitions and facilitate both access to medicines and their quality. In this regard, it is striking that, in a recent WHO survey of counterfeit medicine definitions, of 70 responding countries, 31% had no legal definition of “counterfeit medicine” [44].

Second, the recent seizures of generic medicines in the European Union in transit, under EC Regulation No.1383/2003 [45,46] because of suspicions that they infringed IP law, has caused considerable and understandable concern.

**Medicine quality: difficult or grey areas**

There are several additional important issues that will need to borne in mind during future discussions on the definitions of different types of poor quality medicines.

First, there has been a tendency for poor quality medicines to be considered in terms of the status of the factory producing them—that is, legitimate manufacturers (substandard) versus criminal ‘manufacturers’ (counterfeit). But, because some genuine manufacturers have produced counterfeit medicines, the definition of counterfeit medicine should relate to the end product and not the manufacturer [47].

Second, there is a grey area of overlap between substandard and counterfeit products that depends on the mindset of the manufacturer. Whether there was intent to produce poor quality medicines is the determinant of poor quality type using the 1992 definition of counterfeit medicines (Box1) but not using the proposed new definition (Box 2). The distinction between counterfeit and substandard medicines and the difficulty of deciding whether the ‘intent’ of the manufacturer should be explicitly invoked in definitions of counterfeit medicines raise considerable practical and legal issues and confusion that need further discussion.

Moreover, the production of “substandard” medicines may also involve criminality on the part of legitimate manufacturers. So, for example, there could be intentional production of formulations with insufficient API by legitimate manufacturers, or the quality assurance systems in place at a legitimate factory may be so poor as to warrant punishment through ‘criminal negligence’ or ‘corporate manslaughter’[41]. Thus, if a company buys raw API from a bulk supplier and uses this to produce medicine in good faith but the medicine contains no API, the medicine would be regarded as (disastrously) substandard, but the raw API would be classified as counterfeit. If, however, the bulk supplier intentionally delivered a product containing no API, it would be guilty of counterfeiting whilst the recipient company could be guilty of criminal negligence for not checking the chemical content. Criminality may also be an issue if a manufacturer deliberately varies a formulation without MRA approval, for example, by increasing API concentration because they know that their formulation deteriorates in hot climates. We note with concern that the new definition for substandard medicines recently proposed by WHO [35] omits the statement that genuine medicines are made by manufacturers authorized by NMRAs (Box 2) and would, therefore, include all different types of poor quality medicines. This will mean that, unlike the current definition, counterfeit medicines would also be classed as substandard, as would degraded medicines, and we suggest that this should be reconsidered. The figure in the main text illustrates our suggestion for the relationship between the three different types of poor quality medicine—counterfeit (or synonym, eg falsified), substandard (errors in factory processes) and degraded (degradation of medicine after leaving the factory). This figure reflects the main problems associated with the three types of poor quality medicine and indicates what interventions are required.

Third, if an apparently genuine medicine contains insufficient API it could be substandard because of poor quality production within the factory, or it could be degraded because of high temperatures/humidity post-production. This distinction is very important because the problems that result in substandard and degraded medicines have different origins and solutions. It is currently very difficult to chemically distinguish substandard from degraded medicines and there are remarkably few publicly available data on the chemical and physical changes that medicines undergo in the tropics [48,49].

Finally, there is opposition to the expansion of the definitions for poor quality medicines to include "medical products" such as diagnostic kits and medical devices [20]. This opposition is not in the interests of public health since counterfeit or substandard diagnostic tests and bednets also clearly have the potential to harm public health and safety [3].

**Additional References**

36. Kaur H, Goodman C, Thompson E, Thompson KA, Masanja MI, et al. (2008) A Nationwide survey of the quality of antimalarials in retail outlets in Tanzania. PLoS One 3: e3403.

37. Kelesidis T, Kelesidis I, Rafailidis PI, Falagas ME (2007) [Counterfeit or substandard antimicrobial drugs: a review of the scientific evidence.](http://www.ncbi.nlm.nih.gov/pubmed/17550892) J Antimicrob Chemother 60: 214-236.

# 38. Sengaloundeth S, Green MD, Fernández FM, Manolin O, Phommavong K, et al. (2009) A stratified random survey of the proportion of poor quality oral artesunate sold at medicine outlets in the Lao PDR - implications for therapeutic failure and drug resistance. Mal J 8: 172.

39. Harper J (2006) Counterfeit Medicines. Council of Europe. Strasbourg.

40. Bate R, Mooney L, Harris J, Mitra B (2010) A Safe Medicines Chest for the World. International Policy Press, International Policy Network, London, UK.

41. Cockburn R, Newton PN, Agyarko EK, Akunyili D, White NJ (2005) The global threat of counterfeit drugs: why industry and governments must communicate the dangers. PLoS Medicine 2: e100.

42. Médecins sans Frontières (2010) FATAL FLAWS - How Kenya’s 2008 Anti-Counterfeit Act could endanger access to medicines. Available: http://www.msfaccess.org/content/fatal-flaws-how-kenyas-2008-anti-counterfeit-act-could-endanger-access-medicines. Accessed 10 October 2011.

43. Mullard A (2010) EU implicated in controversial counterfeiting bill. Lancet 375: 1335.

44. World Health Organisation (2010) Preliminary Unedited Draft Survey on National Legislation on “Counterfeit Medicines. Available: <http://www.who.int/entity/medicines/services/counterfeit/WHO_ACM_Report.pdf>. Accessed 10 October 2011.

45. World Trade Organisation (2010) EUROPEAN UNION AND A MEMBER STATE – SEIZURE OF GENERIC DRUGS IN TRANSIT. Request for Consultations by Brazil. WT/DS409/1, IP/D/29, G/L/922, 19 May 2010.

# 46. UNITAID (2009) UNITAID statement on Dutch confiscation of medicines shipment. Available: [**http://www.unitaid.eu/en/resources/news/156-unitaid-statement-on-dutch-confiscation-of-medicines-shipment.html**](http://www.unitaid.eu/en/resources/news/156-unitaid-statement-on-dutch-confiscation-of-medicines-shipment.html). Accessed 10 October 2011.

47. Anon (2009) Pharmaceuticals executives given suspended sentences for counterfeit medicines. MosNews 3^th^ April 2009. Available at: <http://www.mosnews.com/money/2009/04/03/751/>. Accessed 10 October 2011**.**

48. Ballereau F, Prazuck T, Schrive I, Lafleuriel MT, Rozec D et al .(1997) Stability of essential drugs in the field: results of a study conducted over a two-year period in Burkina Faso. Am J Trop Med Hyg 57: 31–36.

49. Keoluangkhot, V, Green M Nyadong L, Fernandez F, Mayxay M et al. (2008) Impaired clinical response in a patient with uncomplicated falciparum malaria who received poor quality and underdosed intramuscular artemether. *Am J Trop Med Hyg* 78: 552-555.
